# An HRM Assay to Differentiate Sheeppox Virus Vaccine Strains from Sheeppox Virus Field Isolates and other Capripoxvirus Species

**DOI:** 10.1038/s41598-019-43158-x

**Published:** 2019-04-30

**Authors:** Tesfaye Rufael Chibssa, Tirumala Bharani K. Settypalli, Francisco J. Berguido, Reingard Grabherr, Angelika Loitsch, Eeva Tuppurainen, Nick Nwankpa, Karim Tounkara, Hafsa Madani, Amel Omani, Mariane Diop, Giovanni Cattoli, Adama Diallo, Charles Euloge Lamien

**Affiliations:** 1 Animal Production and Health Laboratory, Joint FAO/IAEA Agricultural and Biotechnology laboratory, Division of Nuclear Techniques in Food and Agriculture, Department of Nuclear Sciences and Applications, International Atomic Energy Agency, Wagramer Strasse 5, P.O. Box 100, A1400 Vienna, Austria; 2 Institute of Biotechnology, University of Natural Resources and Life Sciences (BOKU), Muthgasse 18, 1190 Vienna, Austria; 3 National Animal Health Diagnostic and Investigation Center (NAHDIC), P.O. Box, 04, Sebeta, Ethiopia; 4 Institute for Veterinary Disease Control, Austrian Agency for Health and Food Safety (AGES), Mödling, Austria; 5 Independent veterinary consultant, Helsinki, Finland; 6 African Union Pan African Veterinary Vaccine Centre, (AU-PANVAC), P.O. Box 1746, Debre Ziet, Ethiopia; 7 Institut National de la Médecine Vétérinaire, Laboratoire Central Vétérinaire d’Alger, Algiers, Algeria; 8 Laboratoire National d’Elevage et de Recherches Vétérinaires, Institut Sénégalais de Recherches Agricoles (ISRA), BP 2057 Dakar-Hann, Dakar, Senegal; 9 UMR CIRAD INRA, Animal, Santé, Territoires, Risques et Ecosystèmes (ASTRE), 24 Montpellier cedex 05, Montpellier, France

**Keywords:** Pox virus, PCR-based techniques

## Abstract

Sheep poxvirus (SPPV), goat poxvirus (GTPV) and lumpy skin disease virus (LSDV) affect small ruminants and cattle causing sheeppox (SPP), goatpox (GTP) and lumpy skin disease (LSD) respectively. In endemic areas, vaccination with live attenuated vaccines derived from SPPV, GTPV or LSDV provides protection from SPP and GTP. As live poxviruses may cause adverse reactions in vaccinated animals, it is imperative to develop new diagnostic tools for the differentiation of SPPV field strains from attenuated vaccine strains. Within the capripoxvirus (CaPV) homolog of the variola virus B22R gene, we identified a unique region in SPPV vaccines with two deletions of 21 and 27 nucleotides and developed a High-Resolution Melting (HRM)-based assay. The HRM assay produces four distinct melting peaks, enabling the differentiation between SPPV vaccines, SPPV field isolates, GTPV and LSDV. This HRM assay is sensitive, specific, and provides a cost-effective means for the detection and classification of CaPVs and the differentiation of SPPV vaccines from SPPV field isolates.

## Introduction

Sheeppox (SPP), goatpox (GTP) and lumpy skin disease (LSD) are three important pox diseases of sheep, goat and cattle respectively^1,2^. The responsible viruses, sheep poxvirus (SPPV), goat poxvirus (GTPV) and lumpy skin disease virus (LSDV) are large, complex, double-stranded DNA viruses of the genus Capripoxvirus, subfamily Chordopoxvirinae, family Poxviridae^3^. Due to the economic importance of the cattle, sheep and goat farming industry and the viruses potential for rapid transboundary spread, SPP, GTP and LSD are categorized by the World Organisation for Animal Health (OIE) as notifiable diseases^4,5^.

SPP and GTP are endemic in Africa, except southern Africa, across the Middle East and Asia^6,7^. SPP incursions have also been reported in Bulgaria and Greece^7^. In early 2018, outbreaks of SPP in Greece went uncontrolled despite implementation of an extensive stamping out campaign^7^. According to the World Animal Health Information System (WAHIS) web portal[WAHID]^8^, the disease is also present in Russia with several sporadic outbreaks reported between 2008 and 2019. The endemic geographic range of LSD was limited to the African continent including Madagascar^2,9^ until recently when the disease emerged in the Middle East before spreading into Asia and Europe. Outbreaks in Albania, Bulgaria, Greece, the Former Yugoslav Republic of Macedonia, Montenegro, Serbia, and the Russian Federation, increase the disease risk for the neighboring countries in the region^7,10^.

Live attenuated SPPV, GTPV and LSDV derived vaccines are widely used in many endemic African, Middle Eastern and Asian countries to control SPP and GTP^11,12^. As live poxviruses may cause adverse reactions in vaccinated animals, it is crucial to use diagnostic tools that can rapidly and specifically differentiate virulent SPPV field isolates from attenuated SPPV vaccine strains. This would help  to elucidate the origin of disease in the event of mild or systemic post-vaccination reactions in vaccinated animals^13^. Similar assays have proven to be very useful in cattle to differentiate the LSDV Neethling vaccine from LSDV fields isolates in Israel^14^, Greece^15^ and Russia^16^.

Routine capripox diagnosis would be improved if one assay was designed to provide both differentiation of SPPV field isolates from SPPV vaccine strains and simultaneously classify the sample into one of three known species: SPPV, GTPV and LSDV. Several tests have addressed the differentiation of SPPV vaccine strains from SPPV field isolates^13,17^, or the genotyping of CaPVs^18–20^, however, a single assay to perform both methods has not been described. This can be accomplished by identifying a suitable genetic marker in the CaPV genome that will allow for the development of a unique, cost-effective test that can be used for both outbreak sample screening and epidemiological investigations in previously vaccinated herds.

With the new generation of dyes and technological improvements in qPCR platforms, high-resolution melting (HRM) curve analysis has become a powerful technique for genotyping microbes, offering the possibility of developing methods other than sequencing for rapid pathogen typing^19,21–24^, including the differentiation of vaccine strains from field isolates^14,16^.

In this paper, we describe the identification of a novel target in the capripox genome and the development of an HRM curve analysis to differentiate SPPV vaccine strains from SPPV field strains and simultaneously identify the three species of CaPVs to support epidemiological surveillance and disease diagnosis during the vaccination period.

## Results

### Assay Design

**Figure 1:**
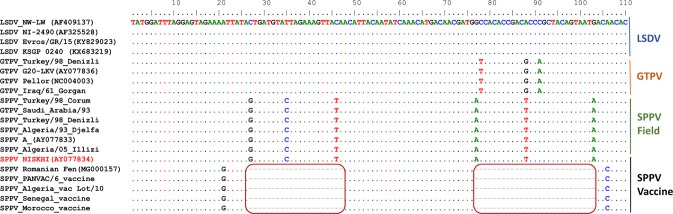
Multiple sequence alignments of partial B22R gene sequences of 20 representative capripoxviruses. Four SPPV vaccine strains, 7 SPPV field strains, 5 GTPV and 4 LSDV field strains were compared. Two series of deletions in the SPPV vaccines (21 and 27 bp) are highlighted in the red boxes. Conserved nucleotides to the reference LSDV sequence are shown as dots while the nucleotide mismatches are represented with the corresponding character.

The analysis of multiple-sequence alignments of CaPV genomes, including a SPPV vaccine from Morocco, enabled the identification of a region with a 21-nucleotide and 27-nucleotide deletion, unique to the SPPV vaccine. This region, within the CaPV homolog of the variola virus B22R gene, also harbored specific nucleotide differences between SPPV field isolates, GTPV and LSDV (Fig. 1). To investigate the uniqueness of these deletions to SPPV vaccines, we sequenced and analyzed the corresponding region in SPPV vaccines from Algeria, Senegal and the Pan African Veterinary Vaccine Center of the African Union Commission (AU-PANVAC). The nucleotide sequences generated in this study have been deposited in GenBank under accession numbers MK005931 to MK005967. The comparative analysis of the nucleotide sequences showed the presence of these two deletions in only the SPPV vaccines, as compared to SPPV field isolates, GTPVs and LSDVs (Supplementary Fig. S1). The sequencing results also confirmed the presence of species-specific nucleotide differences between the SPPV field isolates, GTPV and LSDV. We identified 6 single-nucleotide differences when comparing SPPV field isolates to LSDVs: C/G, T/C, C/T, G/A, A/T, G/A, of which only four nucleotide changes (C/G, G/A, A/T, G/A) can influence the melting temperature. Comparing GTPVs with LSDVs, 7 nucleotide differences were found: G/A, T/A, A/G, C/G, G/A, A/G, G/T, including three aggregate changes that could be exploited during the melting curve analysis: T/A, C/G, G/T.

This region was then targeted to design another set of primers for the amplification of a 110 bp fragment in SPPV vaccine strains and a 158 bp fragment in SPPV field strains, GTPV and LSDV.

Subsequently, we tested the expected melting profiles and melting peaks of the predicted amplicons for SPPV vaccine strains, SPPV field isolates, GTPVs and LSDVs using the uMelt software. The results show four different and distinct melting peaks for SPPV vaccine strains, SPPV field isolates, GTPVs and LSDVs (Supplementary Fig. S2), indicating that the anticipated PCR products could be used to differentiate these four genotypes by HRM.

### PCR Optimization

**Figure 2:**
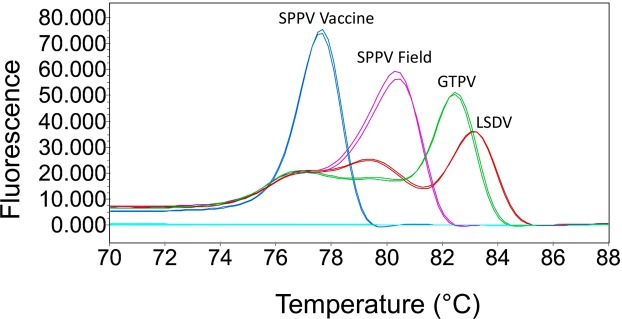
Melting curve variance of SPPV vaccine and three CaPVs Species. The melting curve and melting temperature for SPPV vaccine and each CaPVs species can be well-discerned.

Following an initial testing of a panel of primers, the pair of forward and reverse primers indicated in Table 1 was used for optimization and evaluation of the HRM method. During optimization, the most critical parameters were primer concentration, annealing temperature and time, number of cycles and number of acquisitions during melting. Once the assay was optimized, we evaluated the difference between Tms of all four genotypes using plasmids containing the targeted sequences for SPPV Morocco vaccine, SPPV Turkey/98_Denizli, GTPV Awi/O13/2011 and LSDV Guder/B5/2008. The results show that the SPPV vaccines could be distinctly differentiated from SPPV field isolates and at the same time, the assay could classify CaPV isolates into one of three species (Fig. 2). The average Tm values with the control plasmids (at 10^4^ to 10^6^ copies per reaction) were 76.90 ± 0.13 for SPPV vaccine, 80.27 ± 0.11 for SPPV field isolates, 81.58 ± 0.06 for GTPV and 82.29 ± 0.07 for LSDV.

**Table 1:** Primers used in this study for the HRM assay and for sequencing.

Method	Primer Name	Primer sequence (5′–3′)	Amplicon size
HRM	Cap_ B22RDIV_For	TATGGATTTAGGAGTAGA	158, 110 (Field & vaccine)
	Cap_ B22RDIV_Rev	GCTTTACTTTAATATCATTG	
Sequencing	B22R_ seqHRMFor	TAACGGCATATTGTCTGAATC	250 bp
	B22R_ seqHRMRev	GCTTTACTTTAATATCATTG

### Improved Data Analysis using HRM Software

**Figure 3:**
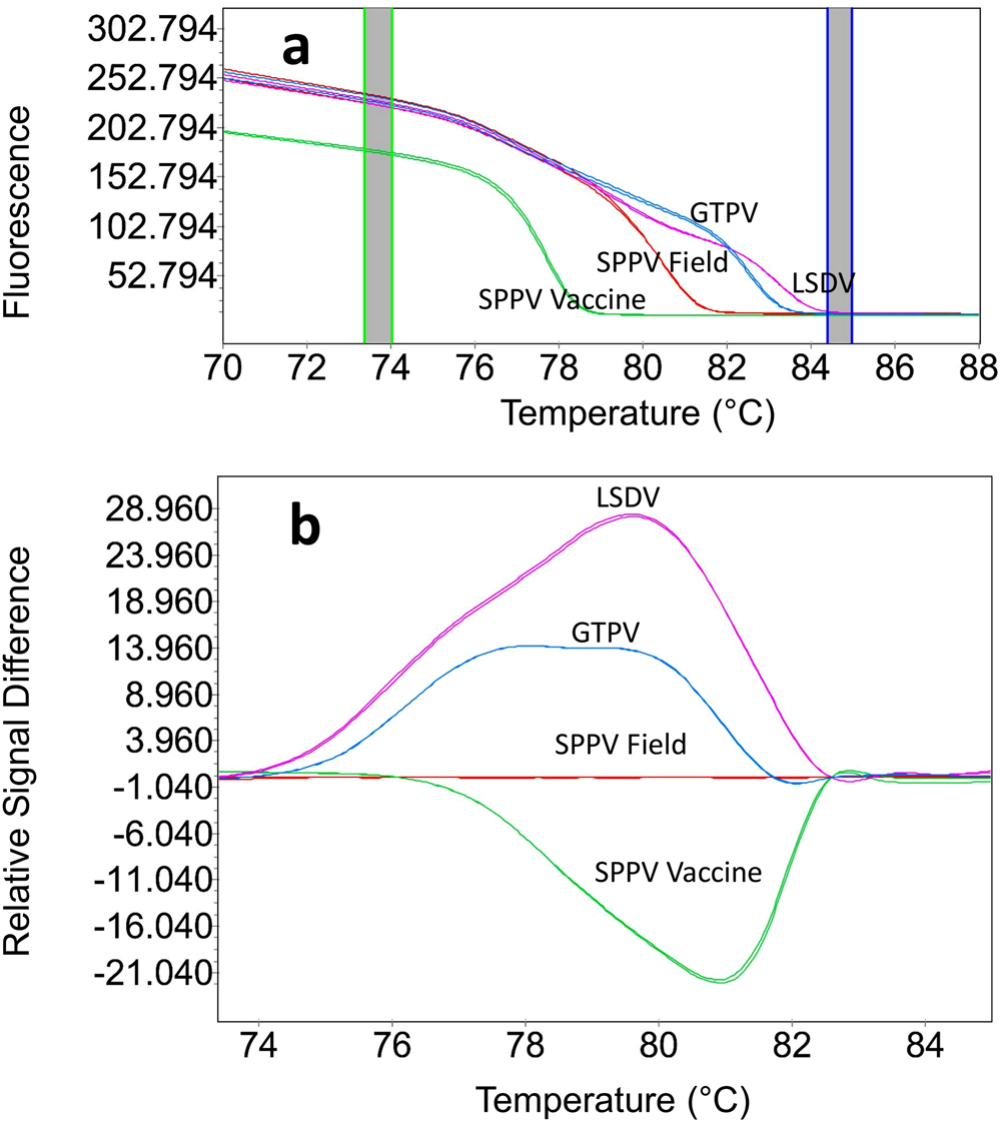
Normalized HRM plots for SPPV vaccine and other CaPVs. (**a**) The graphs visualize the representative profiles of normalized melt curve and (**b**) difference melting curve plots. Each of the genotype species were given a different colored line. The two dark columns in the melting curve plot (**a**) represent pre and post melting normalization regions. In the difference plot (**b**), SPPV field isolates have been selected as the reference for displaying the deviations between SPPV vaccine from GTPV and LSDV field isolates.

To enhance and clarify the data analysis, we used the HRM software to further analyze the melting curves of the PCR amplicons. The HRM analysis of the normalized melting (Fig. 3a) and differential curves (Fig. 3b) were clearly distinct between SPPV vaccine strains, SPPV field isolates, GTPV and LSDV, producing four independent clusters, readily and consistently resolved (Fig. 3a,b). The HRM analysis results were consistent with the conventional melting curve analysis; however, the processed graphs displayed by the HRM software were easier to interpret.

### Limit of Detection of the Assay

The limits of detection, with ≥95% confidence limits, were: 38.04 (31.1–54.14), 17.80 (14.62–26.46), 16.27 (12.63–25.53) and 17.01 (13.22–26.64) copies per reaction for SPPV vaccine, SPPV field isolates, GTPV and LSDV respectively.

### Discriminating Power of the Assay

**Figure 4:**
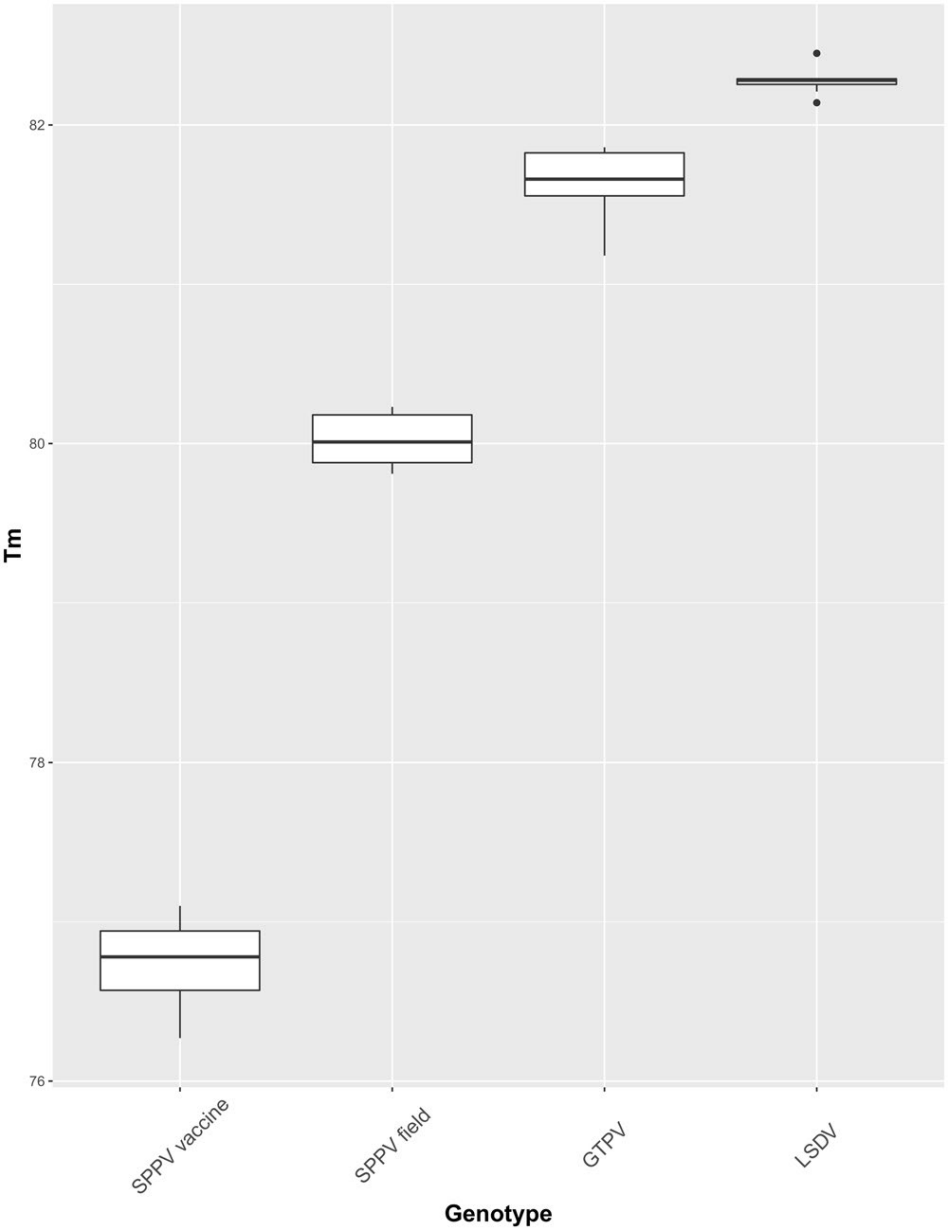
Box plots of the melting temperatures (Tms) of SPPV vaccines and other CaPVs species. The differences in Tm for SPPV vaccine, SPPV field, GTPV and LSDV are shown. The ANOVA test showed a significant difference in average Tm values between the genotypes (P = 0.000).

To study the discriminating power of the HRM assay, 61 DNA samples extracted from SPPV vaccines, cell culture supernatants and clinical samples of CaPVs field isolates, from various geographical locations were screened (Supplementary Table S1). From the 61 samples, we identified SPPV vaccines (n = 4), SPPV field isolates (n = 14), GTPVs (n = 11) and LSDVs (n = 32). The overall Tm ranges, illustrated in Fig. 4, show a clear separation between all four genotypes. The Tm values for the samples were very similar to those of the control plasmids for each genotype: 76.73 ± 0.35 for SPPV vaccine, 80.02 ± 0.16 for SPPV field isolates, 81.66 ± 0.22 for GTPV and 82.29 ± 0.10 for LSDV. One-way ANOVA determined that the average Tm between SPPV vaccines, SPPV field isolates, GTPVs and LSDVs were significantly different (P = 0.000).

We also tested artificial mixtures of control plasmids at 10^4^ copies/µl, at five proportions (1/9, 2/8, 5/5, 8/2 and 9/1) to analyze the potential of the assay to detect coinfection of SPPV vaccine with SPPV field isolates or GTPV or LSDV. At all proportions tested, we observed two readily distinct peaks for the mixture SPPV vaccine/GTPV (at Tm 76.26 for SPPV vaccine and 81.58 for GTPV), and the mixture SPPV vaccine/LSDV (Tm 76.58 for SPPV vaccine and 82.36 for LSDV). In contrast, for the mixture SPPV vaccine/SPPV field produced two readily distinct peaks (Tm 76.38 for SPPV vaccine and 80.25 for SPPV field isolates) at the proportion of 9/1 only. The mixture at the proportion 8/2 presented only a shoulder peak at Tm 76.38 (for the vaccine) and the SPPV field peak at Tm 80.25. The SPPV vaccine peak was absent at other proportions of the mixtures SPPV vaccine/SPPV field. Overall, there was a small shift in the Tm of SPPV vaccine in the mixtures as compared to SPPV vaccine tested alone.

### Specificity

The specificity of the assay was evaluated by testing 13 DNA extracts from other ruminant pathogens (Supplementary Table S2). No amplification was recorded with these non-capripoxvirus pathogens including parapoxviruses.

### Cross-platform Compatibility Test

To assess the cross-platform compatibility of this method, we performed the assay using a selected panel of samples including both plasmid and viral DNA extracted from cell culture supernatants and clinical samples on four different real-time PCR instruments: CFX96 (BioRad), QuantStudio 6 (LifeTechnologies), RotorGene Q (Qiagen) and LightCycler 480 (Roche). This assay was successfully run on all four platforms, utilizing the same mixture and protocol, and succeeded in differentiating SPPV field isolates from SPPV vaccine strains as well as placing the samples in the correct genotypes. (Supplementary Fig. S3). With the various platforms, we noticed a slight shift in the Tm values of the amplicons from one instrument to another (Table 2).

**Table 2:** Cross-platform testing of the HRM assay.

Virus Genotype	Real time PCR machines with Tm values			
	CFX 96 (Bio-Rad)	LC 480 II (Roche)	QS 6 (Life tech)	RG-Q (Qiagen)
SPPV vaccine	75.8–76.3	76.59–76.88	76.45–76.74	77.2–77.54
SPPV field	78.9–79.4	79.87–80.26	79.74–80.22	80.46–80.9
GTPV	80.4–80.4	81.39–81.52	81.29–81.38	82–82.2
LSDV	80.9–80.9	82.07–82.16	81.96–82.06	82.6–82.66

Four different real-time instruments: the LigthCycler 480 (LC 480 II), QuantStudio 6 (QS 6), RotorGene Q (RG-Q) and CFX 96 were used for assay evaluation. The respective amplicon melting temperature values are indicated for SPPV vaccine and other CaPVs.

## Discussion

We have described the identification of specific genetic profiles of CaPV genomes and their use in an HRM-based assay to differentiate SPPV vaccines from SPPV field isolates and further classify CaPVs into SPPV, GTPV or LSDV.

Analysis of the partial fragment of the B22R gene in 37 representative SPPV vaccines, SPPV field isolates, GTPVs and LSDVs available for this study, confirmed two sets of deletions (21 bp and 27 bp) that are specific to the SPPV Romanian and Yugoslavian RM65 vaccine strains, which are used in Morocco, Egypt, Senegal and Algeria and the Romanian Fenner strain (MG000157), which is used as a SPPV vaccine in India. However, the comparisons showed that these deletions are absent in the B22R gene of SPPV NISKHI, an SPPV vaccine used mainly in Russia and countries of the former Soviet Union such as Kazakhstan^25–27^. A previous study reported size differences among CaPVs in the B22R gene and other genes, in the terminal genomic regions, encoding for proteins involved in viral virulence and host range^25^. Although the B22R gene of SPPV NISKHI displayed similarity to virulent SPPVs, prominent differences are present in two of the five CaPV ankyrin repeat-containing genes; a single in-frame stop and a frameshift mutation in SPPV NISKHI SPPV138 and SPPV141, respectively, which resulted into two smaller ORFs, suggesting a different mechanism of attenuation^25,27^ as compared to the SPPV vaccines targeted in this study. Both mutations involved only a single nucleotide and were absent in the draft genome used to design our assay. Similarly, those mutations  were absent in the publicly available genome of SPPV Romanian Fenner (MG000157), a vaccine strain comparable to those tested in this study.

The comparative analysis of the partial B22R sequences of SPPV field isolates, GTPVs and LSDVs generated in this study with those retrieved from full genomes in public databases also confirmed the presence of species-specific signatures that were well-conserved within each of these 3 species, with minor sequence variability noted for both SPPV field isolates and GTPVs (Supplementary Fig. S1). The HRM typing results were in full agreement with sequence results of the PCR amplicons for all 37 samples that were sequenced and the sequence variability had no impact on the melting temperatures within each genotype. Thus, classification of the CaPV isolates into SPPV, GTPV and LSDV using the current HRM assay was consistent with previous methods^18–20^, for all 61 samples in this study. Of interest, is that the assay identified and correctly assigned previously characterized isolates into the correct CaPV species^20^: SPPV KS1, collected from sheep, was identified as a LSDV; SPPV Oman, GTPV Kitengela/O58/2011 and GTPV Kitengela/O59/2011 collected from sheep were identified as a GTPVs; GTPV Saudi Arabia, collected from a goat was identified as a SPPV. Similarly, two recent isolates, GTPV_Awi/O13/2011 and GTPV_Bale/O14/2007, both collected from sheep were identified as GTPVs.

Though we only tested LSDVs from Africa, our sequence analyses showed that LSDVs from Israel (KX894508), Greece (KY829023), Serbia (KY702007) (Supplementary Fig. S1), and Russia (MH893760), were 100% identical to African LSDVs on the targeted fragment of the B22R gene.

Our experiments produced melting curves (Fig. 2) with similar shapes to the uMelt predicted melting curves (Supplementary Fig. S2), with different Tm values, thus illustrating the usefulness of the uMelt simulation software^28^ for the design of HRM-based assays.

The HRM assay displayed good sensitivity, comparable to a previously reported dual-hybridization probe assay for CaPVs’ classification^20^. It was highly specific, with no misidentification of genotypes and no reactivity with other ruminant pathogens tested in this study.

The current HRM assay is easy to perform, interpret and is compatible with various qPCR platforms. Indeed, with all four qPCR instruments tested in this study, the analysis of the melting curves was sufficient to differentiate SPPV vaccines from SPPV field isolates and discriminate between SPPV, GTPV and LSDV. Additionally, we found that using the HRM analysis software enables a better visual representation for the discrimination of different clusters, especially when several samples are analyzed in a single run.

In SPP and GTP endemic countries, the Yugoslavian RM65, the Romanian and the KSGP 0240 strains are the most widely used live attenuated vaccines to protect small ruminants against SPP and GTP^12,13^. The KSGP 0240 vaccine strain which is used in several SPP and GTP endemic countries^12^ was shown to be LSDV^29,30^, therefore, it can be differentiated from SPPV field isolates using species-specific PCR methods^18–20^. The Yugoslavian RM65 strain which is mainly used in the Middle East, Asia and the Horn of Africa^12^, and the Romanian strain, used in India and the Maghreb region^12^, are all SPPV derived vaccines. In the event of capripox outbreaks in small ruminant herds, following the administration of a vaccine based on one of these SPPV strains, it is essential to determine whether the disease is caused by a field isolate or a side effect of the vaccination. Our assay presents the advantage of enabling such a differentiation between SPPV vaccines from SPPV field isolates, and the classification of CaPVs into one of its three species. Two previous reports have described methods to differentiate SPPV vaccines from SPPV field isolates^13,17^, however these methods could not directly classify the CaPV species without additional genotyping methods or sequencing. SPPV and GTPV are not strictly host specific. Similarly, LSDV has been detected in antelope, and the KS1 isolate which was initially collected from small ruminants, has been shown to be LSDV based on sequencing^20,29^. Thus, to implement efficient CaPV disease control measures, it is essential to use specific tools for the accurate identification of circulating isolates in sheep, goats, cattle and wildlife. Indeed, despite the cross-protection displayed by CaPVs, the use of homologous vaccines is preferred as it is believed to provide better protection^7,12^.

Though several assays are available for CaPV species determination by PCR and real time PCR^18–20,30^, none are intended for differentiation of SPPV vaccines from SPPV field isolates. Various research groups developed assays to differentiate the LSDV Neethling vaccine from LSDV field isolates in cattle^14,15^, and furthermore genotype CaPVs^16^; however, those assays are unable to differentiate SPPV vaccines from SPPV field isolates. Therefore, it is expected that this HRM assay will be used as a direct means for screening samples from suspected capripox cases collected in unvaccinated flocks, and to undertake epidemiological investigations when a capripox outbreak occurs following vaccination in a small ruminant or cattle herds.

## Materials and Methods

### Viruses and Nucleic Acid Extraction

A total of 61 CaPV strains were used in this study: four SPPV vaccine strains derived from the Romanian and Yugoslavian RM/65 strains and 57 field isolates of CaPV including cell culture supernatants, and field samples collected from outbreaks at various geographical locations (Supplementary Table S1). In addition, field samples (n = 13) confirmed to be free of CaPV but infected with other ruminant pathogens were used to evaluate the specificity of the new assay (Supplementary Table S2). DNA was extracted from tissue culture supernatant and clinical samples using the AllPrep DNA/RNA extraction kit (Qiagen, Hilden, Germany) following manufacturer’s instructions. The DNA samples were used immediately or kept at −20 °C until use. All pathological samples and viral isolates were handled within the biosafety level 3 facility of the Institute for Veterinary Disease Control, Austrian Agency for Health and Food Safety, Austria.

### Target Gene and Primers Design

In order to identify regions with unique differences between SPPV vaccines and SPPV field isolates, we scanned the sequence alignments of a draft full genome SPPV vaccine from Morocco and representative field isolates and vaccines of all three CaPV species. The sequences were retrieved from Genbank (accession numbers are indicated in Fig. 1) and aligned using MAFFT (http://mafft.cbrc.jp/alignment/server). To broaden the scope of the assay to enable the classification of CaPVs, we selected the final region based on the following: (1) the selected region offered markers for an unequivocal differentiation of SPPV vaccines, derived from the Romanian and Yugoslavian RM65, from SPPV field isolates; (2) the region presented enough nucleotide differences between the SPPVs, GTPVs and LSDVs that enabled the separation of these three species by HRM; (3) the differences between SPPV, GTPV and LSDV were well conserved within each CaPV species, thus representing strong species-specific signatures for genotyping. A region within the B22R gene of CaPVs which contains two separate nucleotide deletions of 21 and 27 nucleotides in length was selected. The same region also presented species-specific signatures for SPPV, GTPV and LSDV. A primer pair (Table 1) flanking this region was selected (positions 122045 to 122202 in SPPV A genome) to amplify and sequence the targeted regions in all SPPV vaccines, and in some representatives of SPPV field isolates, GTPV and LSDV available in our laboratory. Another set of primers (positions 121952 to 122202 in SPPV A genome) was designed for evaluation in the HRM assay (Table 1). To confirm the suitability of these primers in the HRM assay to differentiate SPPV vaccines from SPPV field isolates with the simultaneous classification of CaPVs, we performed an in-silico simulation, with the predicted PCR amplicons for SPPV vaccines, SPPV field isolate, GTPV and LSDV, using the uMelt software^28^. uMelt is a flexible web-based tool for predicting DNA melting curves and denaturation profiles of PCR products. The above-mentioned primers were selected using Allele ID version 6 software (Premier Biosoft International, Palo Alto, CA, USA), synthesized and purified by reverse phase high-performance liquid chromatography by Eurofins, Germany. The specificity of the primer sequences were checked using the Basic Local Alignment Search Tool (NCBI/Primer-BLAST). From now on, we will refer to each of these four groups, SPPV vaccines, SPPV field isolates, GTPVs and LSDVs, as genotypes.

### Positive Controls

Positive control plasmids, containing the target gene fragment of the B22R gene of SPPV Morocco vaccine, SPPV Turkey/98 Denizli, GTPV Awi/O13/2011 and LSDV Guder/B5/2008 isolates were constructed by ligating the respective PCR products into the pGEM-T Easy Vector System (Promega, Madison, WI, USA) as per the manufacturer’s instructions. The ligated products were used to transform E. coli DH5α competent cells (Invitrogen, Carlsbad, CA, USA). The positive clones were propagated, and the plasmid DNA extracted using the PureYield plasmid Midiprep System (Promega, Madison, WI, USA). The purified plasmids were quantified with the Nanodrop 3300 Fluorospectrometer using the PicoGreen dsDNA reagent kit (Thermo Fisher Scientific, Waltham, MA, USA) per manufacturer’s instructions and sequenced to confirm the presence of the correct insert. The copy number was calculated as described by Lamien *et al*.^20^.

### HRM Analysis

The 10 µl PCR reaction mixture contained 1X Lightscanner master mix (BioFire Defense, Utah, USA.), 250 nM of each primer (Cap_ B22RDIV_For and Cap_ B22RDIV_Rev) and 2 µl of sample DNA. The PCR reaction was performed in a LightCycler 480 II Real time PCR Detection System (Roche Diagnostics Corporation, IN, USA) with an initial denaturation at 95 °C for 4 min, followed by 42 cycles of 95 °C for 5 sec, 58 °C for 5 sec and 72 °C for 5 sec. The PCR products were then denatured at 95 °C for 30 sec, cooled to 65 °C for 1 min, and melted from 65 °C to 90 °C at a rate of 100 acquisitions per °C. For each set of reactions, positive control plasmids and negative controls consisting of nuclease-free water in place of the template DNA were included. High Resolution Melting (HRM) curve analysis was performed using the LightCycler 480 Gene Scanning Software (Roche) to analyze the data and melting profiles of SPPV vaccines, SPPV field isolates, GTPVs and LSDVs. Normalized melt curves and difference plots were obtained by analyzing the active melt region and designating the pre and post melt regions.

### Limit of Detection

Each plasmid was 10-fold serially diluted from 10^7^ to 10^1^ viral copies/µL using Herring sperm DNA matrix (5 ng/mL) and kept at 4 °C until further analysis. First, the linearity of the assay was analyzed for each viral species to determine the efficiency and dynamic range of the assay. Subsequently, the limit of detection (LOD) of the method was assessed by amplifying eight different concentrations (100, 80, 60, 40, 20, 10, 5 and 0 viral copies/µL) of each plasmid, corresponding to each of SPPV vaccine, SPPV field isolate, GTPV and LSDV. Five replicates of different plasmid concentrations were tested on four separated occasions, and the proportion of positive results were determined for each concentration. The LOD of the current assay was determined by probit regression analysis.

### Discriminating Power and Specificity of the Assay

The discriminating power of the developed HRM assay was evaluated using DNA (n = 61) extracted from suspected capripox clinical samples and CaPV infected cell culture suspensions from sheep, goats and cattle from different geographical regions (Supplementary Table S1). The accuracy of the assay results was confirmed by comparing the HRM genotyping results with the sequencing data of the amplicons. The specificity of the method was also evaluated by testing DNA (n = 13) extracted from Mycoplasma mycoides subsp. mycoides Small Colony (MmmSC), Orf virus (ORFV), Bovine herpes virus 1 and 2, and cDNA from Peste des Petits Ruminants virus (PPRV), (Supplementary Table S2).

### Statistical Analysis

All statistical analyses were performed in R (version 3.4.1) via Rstudio (version 1.1.383). A One-Way ANOVA test and Tukey multiple comparisons of means were performed in R to determine whether the average Tm differences between the genotypes were significant. In addition, box and whisker plots were constructed to illustrate the differences between the Tm of the four genotypes using the ggplot2 package in R. For LOD determination, the LC_probit function of the ecotox package in R was used to calculate the predicted limits of detection and their confidence limits (CL) using a probit analysis. The dose-response curves were plotted using ggplot2 in R.

### Cross Platform Compatibility Test

The cross-platform compatibility of this assay was evaluated using various real time PCR instruments. Thus, using the same PCR mix and protocol as in the LightCycler 480 II (Roche) instrument, PCR and melting curve acquisition analysis was also conducted using the CFX96 Real Time PCR system (Bio-Rad, Hercules, CA, USA), QuantStudio 6 Flex Real Time PCR system (ThermoFisher Scientific Inc., Waltham, MA, USA), and Rotor Gene Q Real Time PCR cycler (Qiagen).

### Nucleotide Sequencing and Analysis

A 250 bp region of the B22R gene containing the targeted HRM fragment was amplified by PCR for selected samples, using the primers B22R seqHRM-For and B22R- seqHRM-Rev (Table 1), and sequenced. The 20 μl PCR reaction mixture contained 500 nM of each primer, 0.2 mM dNTPs, 2.5U Taq DNA polymerase (Qiagen), 1X PCR buffer, and 2 μl template DNA. The cycling conditions were as follows: 95 °C for 4 min, followed by 35 cycles at 95 °C for 30 sec. 58 °C for 30 sec, and 72 °C for 30 sec, and a final extension at 72 °C for 2 min. PCR products were cheeked by electrophoresis on a 2% agarose gel for 1 h at 100 V. The PCR products were purified using Wizard SV Gel and PCR Clean Up System (Promega) and sequenced commercially by LGC Genomics (Germany). The sequence data were edited, and the fragments were assembled using Vector NTI 11.5 software (Invitrogen). Additional nucleotide sequences of the corresponding regions for SPPV, GTPV and LSDV isolates were retrieved from GenBank for comparative analysis. Multiple sequence alignments were performed using the CLUSTALW algorithm implemented in BioEdit 7.5 software package.

## Supplementary information

Supplementary file

## Data Availability

DNA sequences have been deposited in GenBank and the accession numbers were provided in the manuscript. All the remaining datasets generated during this study are available from the corresponding author on request.
