# Supplementary material for: An HRM Assay to Differentiate Sheeppox Virus Vaccine Strains from Sheeppox Virus Field Isolates and other Capripoxvirus Species
Source: Sci Rep. 2019 Apr 30;9:6646. doi: 10.1038/s41598-019-43158-x (PMC6491823; doi:10.1038/s41598-019-43158-x)
Supplement: Supplementary file 1 — Supplementary file [file 41598_2019_43158_MOESM1_ESM.docx]

**An HRM Assay to Differentiate Sheeppox Virus Vaccine Strains from Sheeppox Virus Field Isolates and other Capripoxvirus Species**

Tesfaye Rufael Chibssa ^1,2,3^, Tirumala Bharani K Settypalli ^1^, Francisco J. Berguido ^1^, Reingard Grabherr ^2^, Angelika Loitsch ^4^, Eeva Tuppurainen ^5^, Nick Nwankpa ^6^, Karim Tounkara ^6^, Hafsa Madani ^7^, Amel Omani ^7^, Mariane Diop ^8^, Giovanni Cattoli ^1^, Adama Diallo ^8,9^ and Charles Euloge Lamien ^1,^*

^1^ Animal Production and Health Laboratory, Joint FAO/IAEA Agricultural and Biotechnology laboratory, Division of Nuclear Techniques in Food and Agriculture, Department of Nuclear Sciences and Applications, International Atomic Energy Agency, Wagramer Strasse 5, P.O. Box 100, A1400, Vienna, Austria

^2^ Institute of Biotechnology, University of Natural Resources and Life Sciences (BOKU), Muthgasse 18, 1190 Vienna, Austria

^3^ National Animal Health Diagnostic and Investigation Center (NAHDIC), P.O Box, 04, Sebeta, Ethiopia;

^4^ Institute for Veterinary Disease Control, Austrian Agency for Health and Food Safety (AGES), Mödling, Vienna,

^5^ Independent veterinary consultant

^6^ African Union Pan African Veterinary Vaccine Centre, (AU-PANVAC) P. O. Box 1746, Debre Ziet, Ethiopia;

^7^ Institut National de la Médecine Vétérinaire, Laboratoire Central Vétérinaire d’Alger, Algiers, Algeria

^8^ Laboratoire National d'Elevage et de Recherches Vétérinaires, Institut Sénégalais de Recherches Agricoles (ISRA), BP 2057 Dakar-Hann, Dakar, Sénégal

^9^ UMR CIRAD INRA, Animal, Santé, Territoires, Risques et Ecosystèmes (ASTRE), 24 Montpellier cedex 05, France

***** Correspondence: C.Lamien@iaea.org; Tel.: +43-1-2600-28314 (C.E.L.)

**Supplementary information**

**Supplementary table S1**. Capripoxviruses tested by the HRM for this study.

|  | **Isolate name** | **Origin** | **Year** | **Source** | **Type of samples** | **Host** | **Tm** | **Genotype** |
| --- | --- | --- | --- | --- | --- | --- | --- | --- |
| 1 | LSDV_Egypt/98_Ismailia | Egypt | 1998 | HSL-AGES | CC | Cattle | 82.27 | LSDV |
| 2 | LSDV_Ziway/B3/2011 | Ethiopia | 2011 | NAHDIC | SL | Cattle | 82.27 | LSDV |
| 3 | LSDV PANVAC-7 | Egypt | NA | PANVAC | CC | Cattle | 82.27 | LSDV |
| 4 | SPPV_KS-1 | Kenya | 1976 | HSL-AGES | CC | Sheep | 82.14 | LSDV |
| 5 | LSDV Marsabit/B291/2007 | Kenya | 2007 | Kenya | SL | Cattle | 82.21 | LSDV |
| 6 | LSDV Arsi/B1/2011 | Ethiopia | 2011 | NAHDIC | SL | Cattle | 82.21 | LSDV |
| 7 | LSDV Sudan North/2011 | Sudan | 2011 | CVRL/Sudan | SL | Cattle | 82.14 | LSDV |
| 8 | LSDV Sundus/2012 | Sudan | 2012 | CVRL/Sudan | SL | Cattle | 82.14 | LSDV |
| 9 | LSDV_Toke/B6/2008 | Ethiopia | 2008 | NAHDIC | SL | Cattle | 82.27 | LSDV |
| 10 | LSDV Asella/B2/2011 | Ethiopia | 2011 | NAHDIC | DNA | Cattle | 82.21 | LSDV |
| 11 | LSDV Adama/B4/2011 | Ethiopia | 2011 | NAHDIC | DNA | Cattle | 82.21 | LSDV |
| 12 | LSDV Ambo/B8/2008 | Ethiopia | 2008 | NAHDIC | DNA | Cattle | 82.27 | LSDV |
| 13 | LSDV chilimo/B11/2008 | Ethiopia | 2008 | NAHDIC | DNA | Cattle | 82.27 | LSDV |
| 14 | LSDV Galesa/B12/2008 | Ethiopia | 2008 | NAHDIC | DNA | Cattle | 82.27 | LSDV |
| 15 | LSDV Humbo/B23/2010 | Ethiopia | 2010 | NAHDIC | DNA | Cattle | 82.21 | LSDV |
| 16 | LSDV Sodo/B24/2010 | Ethiopia | 2010 | NAHDIC | DNA | Cattle | 82.27 | LSDV |
| 17 | LSDV Sennar/12 | Sudan | 2012 | CVRL | SL | Cattle | 82.45 | LSDV |
| 18 | LSDV_BG24/2010 | Kenya | 2010 | Kenya | SS | Cattle | 82.29 | LSDV |
| 19 | LSDV_B291 /2007 | Kenya | 2007 | Kenya | SL | Cattle | 82.45 | LSDV |
| 20 | LSDV_B338 / 2011 | Kenya | 2011 | Kenya | SL | Cattle | 82.29 | LSDV |
| 21 | LSDV_Massalamia P4 | Sudan | 1971 | Sudan | CC | Cattle | 82.45 | LSDV |
| 22 | LSDV_Massalamia P66 | Sudan | NA | Sudan | CC | Cattle | 82.29 | LSDV |
| 23 | LSDV_MB597 LN AMB C1 | Ethiopia | 2014 | NVI | LN | Cattle | 82.45 | LSDV |
| 24 | LSDV_MB575 /14 NS K24 | Ethiopia | 2014 | NVI | NS | Cattle | 82.45 | LSDV |
| 25 | LSDV_Mojo BG cattle | Ethiopia | 2011 | NVI | SL | Cattle | 82.45 | LSDV |
| 26 | LSDV_MB581 TS Fic 7715 | Ethiopia | 2014 | NVI | SL | Cattle | 82.29 | LSDV |
| 27 | LSDV_Wonji S Cattle | Ethiopia | 2011 | NVI | SL | Cattle | 82.29 | LSDV |
| 28 | LSDV_MB589 TS Qur C1 | Ethiopia | 2014 | NVI | SL | Cattle | 82.29 | LSDV |
| 29 | LSDV_Akaki TS C-1 | Ethiopia | 2014 | NVI | SL | Cattle | 82.29 | LSDV |
| 30 | LSDV_MB581 TS Fic 7758 | Ethiopia | 2014 | NVI | SL | Cattle | 82.45 | LSDV |
| 31 | LSDV_MB607 TS Tig C3 | Ethiopia | 2014 | NVI | SL | Cattle | 82.29 | LSDV |
| 32 | LSDV_MB597 NS Am C9 | Ethiopia | 2014 | NVI | NS | Cattle | 82.29 | LSDV |
| 33 | GTPV_Iraq/61_Gorgan | Iraq | 1961 | Pirbright | CC | Goat | 81.79 | GTPV |
| 34 | GTPV_Mongolia/C3/08 | Mongolia | 2008 | Mongolia | SL | Goat | 81.86 | GTPV |
| 35 | GTPV_Oman/84 | Oman | 1984 | Pirbright | CC | Goat | 81.86 | GTPV |
| 36 | GTPV_Awi/O13/2011 | Ethiopia | 2011 | NAHDIC | SL | sheep | 81.66 | GTPV |
| 37 | GTPV_Bale/O14/2007 | Ethiopia | 2007 | NAHDIC | SL | sheep | 81.66 | GTPV |
| 38 | GTPV_Ghana | Ghana | 2011 | Pirbright | CC | Goat | 81.18 | GTPV |
| 39 | GTPV Kitengela/O58/2011 | Kenya | 2011 | CVL | SL | sheep | 81.79 | GTPV |
| 40 | GTPV_Yemen/83 | Yemen | 1983 | Pirbright | CC | Goat | 81.45 | GTPV |
| 41 | SPPV_Oman/84 | Oman | 1984 | Pirbright | CC | Goat | 81.45 | GTPV |
| 42 | GTPV Kitengela/O59/2011 | Kenya | 2011 | CVL | SL | sheep | 81.66 | GTPV |
| 43 | Mog/ GP/T5/2008 | Mongolia | 2008 | Mongolia | SS | Goat | 81.86 | GTPV |
| 44 | SPPV_Mongolia_/O2/07 | Mongolia | 2007 | Mongolia | SL | sheep | 79.88 | SPPV |
| 45 | SPPV_Mauritania/85_Gorgol | Mauritania | 1985 | Mauritania | SL | Sheep | 80.18 | SPPV |
| 46 | SPPV_Mongolia/T1/06 | Mongolia | 2006 | Mongolia | SL | sheep | 79.88 | SPPV |
| 47 | SPPV Turkey/98 Van 2 | Turkey | 1998 | VCRI-Pendik | CC | sheep | 80.23 | SPPV |
| 48 | SPPV_Turkey/98_Corum | Turkey | 1998 | VCRI-Pendik | CC | Sheep | 79.88 | SPPV |
| 49 | GTPV_Saudi_Arabia/93 | Saudi Arabia | 1993 | Pirbright | CC | Goat | 80.01 | SPPV |
| 50 | SPPV_Turkey/98_Darica | Turkey | 1998 | VCRI-Pendik | CC | Sheep | 80.01 | SPPV |
| 51 | SPPV_Turkey/98_Denizli | Turkey | 1998 | VCRI-Pendik | CC | Sheep | 80.23 | SPPV |
| 52 | SPPV_Algeria/93_Djelfa | Algeria | 1993 | INMV-LCV | CC | Sheep | 79.81 | SPPV |
| 53 | SPPV_HSL | Unknown | NA | HSL-AGES | SL | sheep | 80.23 | SPPV |
| 54 | SPPV_Algeria/05_Illizi | Algeria | 2005 | INMV-LCV | CC | Sheep | 80.18 | SPPV |
| 55 | SPPV Niger/88 | Niger | 1988 | CIRAD | CC | Sheep | 80.01 | SPPV |
| 56 | Mog/SP/T3/2007 | Mongolia | 2007 | Mongolia | SS | sheep | 79.81 | SPPV |
| 57 | SPPV_Turkey/98_Sivas | Turkey | 1998 | VCRI-Pendik | CC | Sheep | 79.88 | SPPV |
| 58 | SPPV PANVAC-6 Vaccine | Egypt | NA | PANVAC | CC | sheep | 76.27 | SPPV vaccine |
| 59 | SPPV_Algeria_vaccine_Lot_7 | Algeria | 2009 | INMV-LCV | CC | Sheep | 76.67 | SPPV vaccine |
| 60 | SPPV_Senegal_vaccine | Senegal | NA | CIRAD | CC | Goat | 76.89 | SPPV vaccine |
| 61 | SPPV_Morocco_vaccine | Morocco | NA | Biopharma | CC | Sheep | 77.1 | SPPV vaccine |

Abbreviations: VCRI = Veterinary Control and Research Institute; LNERV-ISRA = Laboratoire National de l'Elevage et de Recherches Vétérinaires, Institut Sénégalais de Recherches Agricoles ; INMV-LCV = Institut National de la Médecine Vétérinaire, Laboratoire Central Vétérinaire; IVM = Institute of Veterinary Medicine; Pirbright = The Pirbright Institute; PANVAC = Pan African Veterinary Vaccine Centre; NAHDIC = National Animal Health Diagnostic and Investigation Center; HSL-AGES = High Security Laboratory, Austrian Agency for Health and Food Safety; CVL: Central Veterinary Laboratories. NA = Not Applicable; CC = Cell culture; SL = Skin lesions; SC = Skin scraping; NS = Nasal swab; LN = Lymph node

**Supplementary table S2**. Non-capripoxvirus samples used for this study.

| **No.** | **Strain name** | **Source** | **Country** | **Sample type** | **Host** |
| --- | --- | --- | --- | --- | --- |
| 1 | ORFV MB38/13 C-2 | NVI/Ethiopia | Ethiopia | Skin scraping | Sheep |
| 2 | ORFV MB38/13 C-3 | NVI/Ethiopia | Ethiopia | Skin scraping | Sheep |
| 3 | ORFV MB38/13 C-4 | NVI/Ethiopia | Ethiopia | Skin scraping | Sheep |
| 4 | ORFV MB38/13 C-5 | NVI/Ethiopia | Ethiopia | Skin scraping | Goat |
| 5 | ORFV MB38/13 C-6 | NVI/Ethiopia | Ethiopia | Skin scraping | Goat |
| 6 | ORFV MB38/13 C-7 | NVI/Ethiopia | Ethiopia | Skin scraping | Goat |
| 7 | PPRV (cDNA) | NVI/Ethiopia | Ethiopia | Nasal swab | Goat |
| 8 | PPRV (cDNA) | NVI/Ethiopia | Ethiopia | Nasal swab | Goat |
| 9 | PPRV (cDNA) | NVI/Ethiopia | Ethiopia | Nasal swab | Goat |
| 10 | Mccp | NVI/Ethiopia | Ethiopia | Pathological lesions | Goat |
| 11 | Mccp | NVI/Ethiopia | Ethiopia | Pathological lesions | Goat |
| 12 | BOHV-1 404/2018 | HSL-AGES/Austria | Austria | Cell culture | Cattle |
| 13 | BOHV-2 95-5/2016 | HSL-AGES/Austria | Austria | Cell culture | Cattle |

Abbreviations: BOHV = Bovine herpes virus; Mccp= Mycoplasma capricolum ssp. Capripneumonia; NVI = National Veterinary Institute; HSL-AGES = High Security Laboratory, Austrian Agency for Health and Food Safety; PPRV (cDNA) = Peste des petits ruminants virus complementary deoxyribonucleic acid; ORFV= Orf Virus


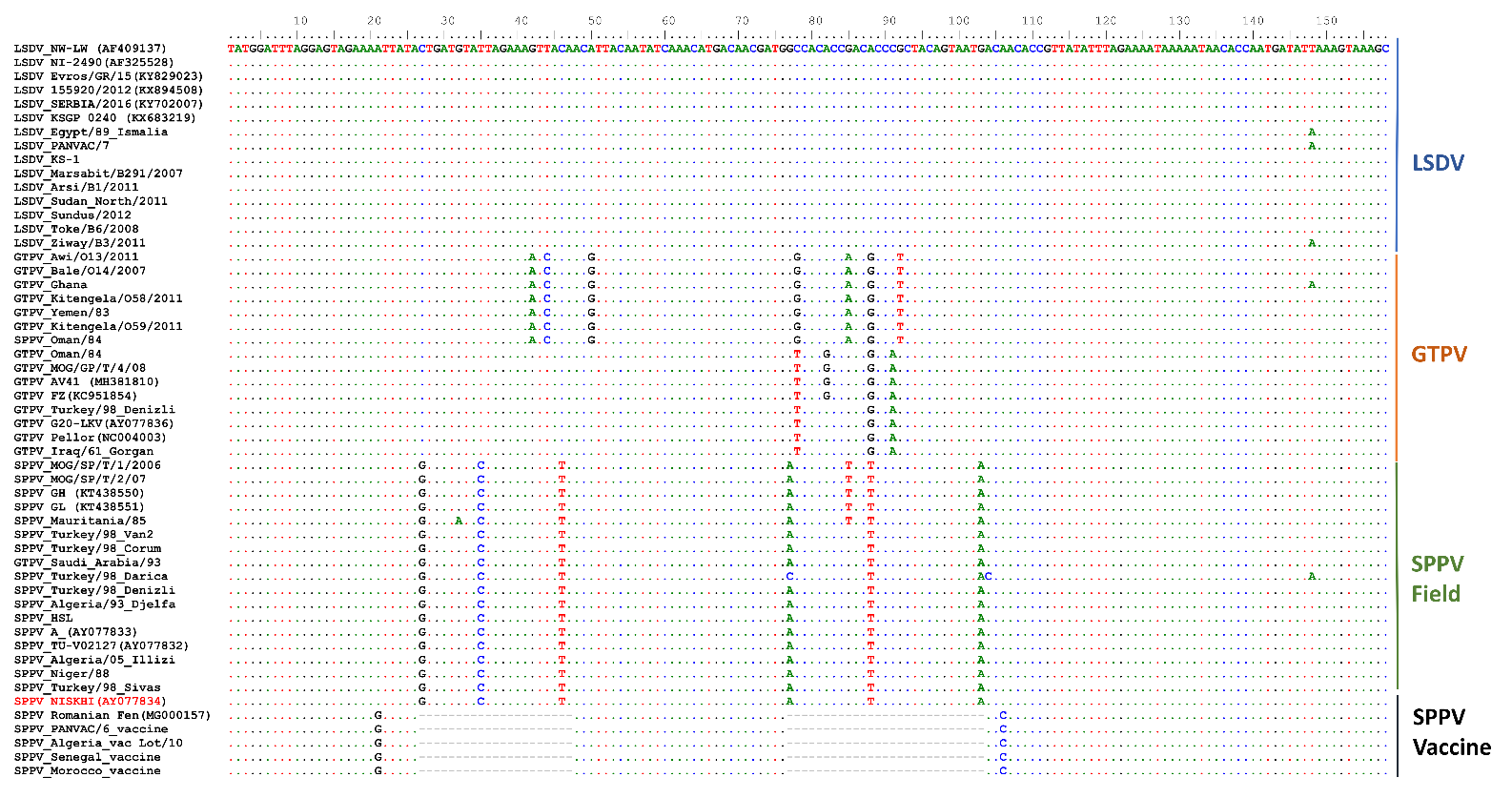


**Supplementary Figure S1.** Multiple sequence alignments of the partial B22R gene sequences of 37 capripoxviruses from this study and representative capripoxviruses from GenBank.


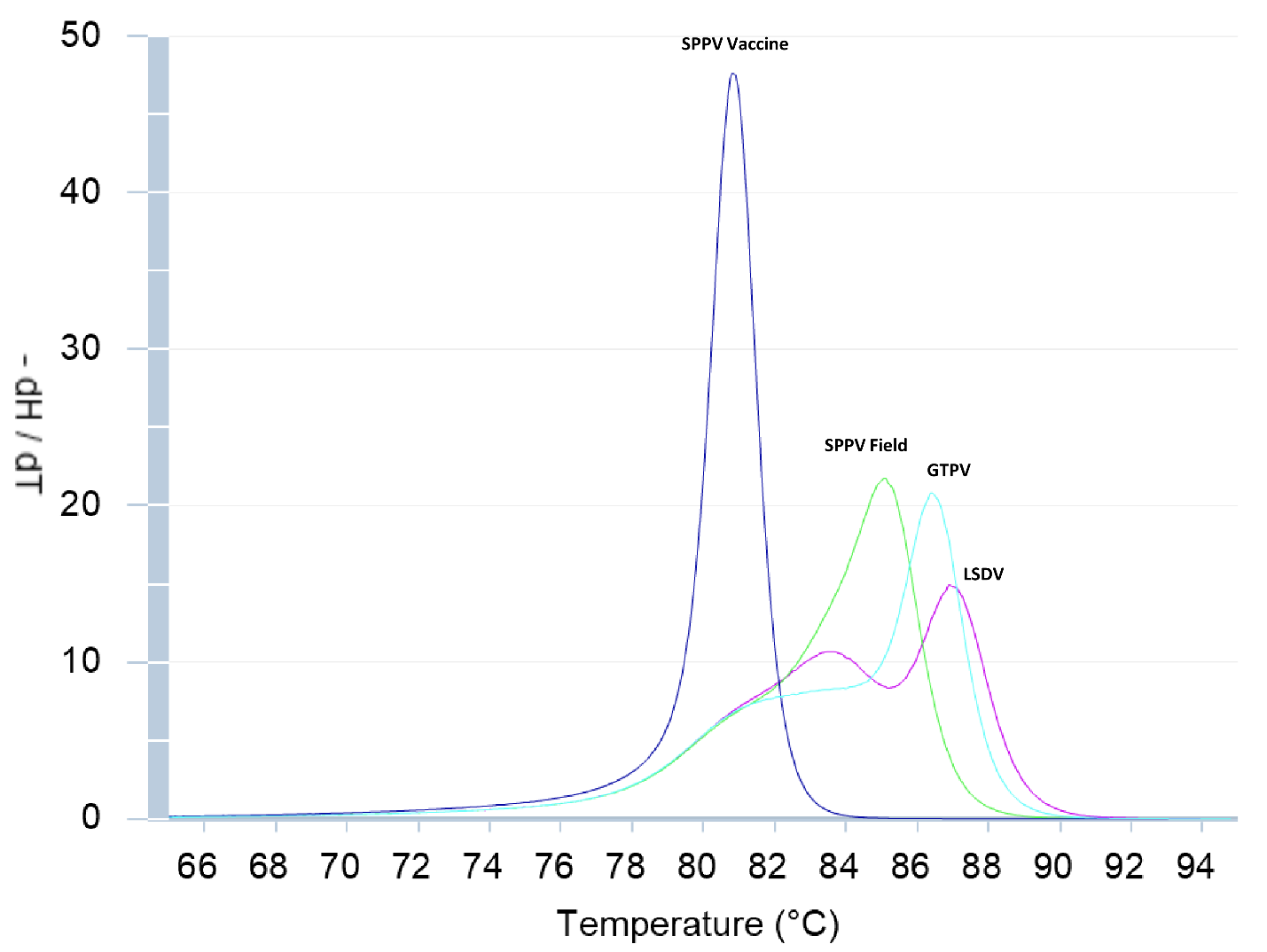


**Supplementary Figure S2.** Derivative melt curves, using the uMELT BATCH2.2, for SPPV vaccine, SPPV field isolate, GTPV and LSDV. Sugimoto (Nucleic Acids, 1996) thermodynamic library was used, with a temperature range from 65 to 95°C. Resolution setting was 0.1°C and salt concentrations were 20 mM for Mono+ and 3 mM for Mg++. The results show 4 distinct melting peaks for SPPV vaccine (Tm = 80.8°C), SPPV field isolate (Tm = 85.0°C), GTPV (Tm = 86.4°C) and LSDV (Tm = 87.1°C)


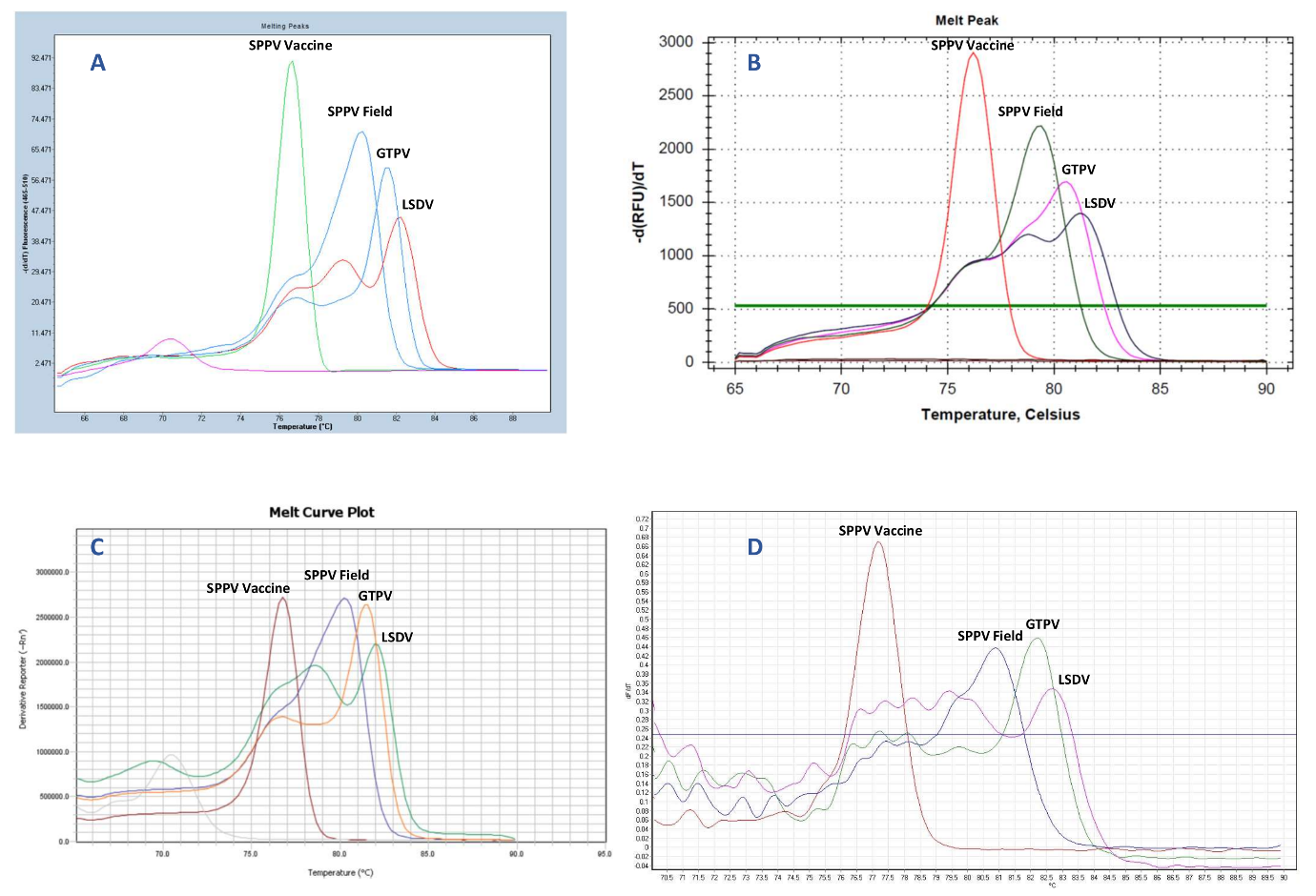


**Supplementary Figure S3.** Cross-platform testing of the HRM assay. The melting curves of SPPV vaccine, SPPV field isolate, GTPV and LSDV are shown for: (A) the LightCycler 480 II (Roche), (B) the CFX96 Real Time PCR system (Bio-Rad), (C) the QuantStudio 6 Flex Real Time PCR system (ThermoFisher Scientific Inc) and (D) the Rotor Gene Q Real Time PCR cycler (Qiagen).
